# A rare presentation of *Klebsiella pneumoniae* endogenous panophthalmitis with optic neuritis and orbital cellulitis from a urinary tract infection

**DOI:** 10.1016/j.idcr.2021.e01289

**Published:** 2021-09-23

**Authors:** Soumaya Bouhout, Magaly Lacourse, Annie-Claude Labbé, Marie-Josée Aubin

**Affiliations:** a Department of Ophthalmology, Université de Montréal, Montréal, Québec, Canada; b Department of Microbiology, Infectious Diseases and Immunology, Université de Montréal, Montréal, Québec, Canada; c Division of Infectious Diseases and Microbiology, Hôpital Maisonneuve-Rosemont, CIUSSS de l’Est-de-l’Île-de-Montréal, Montréal, Québec, Canada; d Centre universitaire d’ophtalmologie (CUO), Hôpital Maisonneuve-Rosemont (HMR), CIUSSS de l’Est-de-l’Île-de-Montréal, Montréal, Québec, Canada; e Department of Social and Preventive Medicine, School of Public Health, Université de Montréal, Montréal, Québec, Canada

## Abstract

This case illustrates the rare presentation of endogenous *Klebsiella pneumoniae* endophthalmitis with concomitant orbital cellulitis from an acute pyelonephritis. A 59-year-old Caucasian female with type 2 diabetes mellitus was transferred from a regional hospital with decreased visual acuity, periorbital edema, photophobia, proptosis and pain of the right eye, as well as suprapubic discomfort. Initial ocular examination and B-scan ultrasonography were consistent with endophthalmitis and orbital cellulitis which lead to a vitreous tap and intravitreal antibiotics injection and systemic antibiotherapy. Vitreous and blood cultures confirmed *Klebsiella pneumoniae* as the causative organism. An orbital MRI showed a panophthalmitis with optic neuritis and further imaging confirmed a concomitant pyelonephritis secondary to a septic nephrolithiasis. The patient was given treatment with high-does intravenous antibiotics, oral and topical corticotherapy, and an early core pars plana vitrectomy (PPV), performed 5 days after presentation with repeat injections of antibiotics and dexamethasone. Unfortunately, two weeks following PPV, despite an initial stable postoperative course, the patient deteriorated and presented with purulent discharge from one of the vitrectomy port incision site. An emergency evisceration was performed in order to control the infection, revealing a large subretinal abscess and necrosed sclerotic tissue around the prior vitrectomy incision sites. Conclusion: This is the first case report of *Klebsiella pneumoniae* endophthalmitis or panophthalmitis presenting with orbital cellulitis and optic neuritis from an urinary tract infection. Prognosis is poor despite treatment including early vitrectomy.

## Introduction

Klebsiella species are facultative anaerobic Gram-negative bacilli, which can be part of the gastrointestinal and nasopharyngeal flora [1]. They are highly prevalent causative organisms for endogenous endophthalmitis (EE) in Asia (3–37%) and is often associated with a pyogenic liver abscess and diabetes mellitus [1]. By contrast, it is relatively rare in North America where it accounts for only 3.6% of endogenous endophthalmitis [1]. Indeed, most endogenous endophthalmitis in North America and Europe are caused by Streptococci (30–50%), other Gram-negative bacilli (30%) and *Staphylococcus aureus* (25%)[2].

As seen in Table 1, there are only few reported cases of endogenous Klebsiella panophthalmitis [3], [4] or endophthalmitis [5], [6], [7] with concomitant orbital cellulitis. This case report describes the first documented case of Klebsiella endogenous panophthalmitis, with associated optic neuritis and orbital cellulitis secondary to a urinary tract infection (UTI).

**Table 1:** Review of the literature of endogenous Klebsiella optic neuritis or panophthalmitis and endophthalmitis with orbital cellulitis. AC: anterior chamber. DM: Diabetes mellitus. EVI: endoscopic variceal injections. IV: intravenous. IVDU: intravenous drug use. HM: Hand motion. LP: Light perception. NA: not available. OHT: ocular hypertension PPV: pars plana vitrectomy. RAPD: Relative afferent pupillary defect. RD: retinal detachment*.*

**Tyee of presentation**	**Case**	**Age/Sex**	**Underlying medical conditions**	**Systemic infection foci**	**Initial symptoms**	**Initial VA**	**Ocular signs**	**Orbital Imaging**	**Medical management**	**Pars Plana Vitrectomy performed (timing / findings)?**	**Complications**	**Final VA (timing)**
**Endophthalmitis/panophthalmitis and orbital cellulitis**	1 [*Hung* et al.^*2*^]	66 M	Liver cirrhosis	Translocation from EVI	Fever, chills, decreased VA, ocular pain	NA	Conjunctiva chemosis, edematous cornea, shallow AC with hypopyon, no vitreous view	CT scan: abnormal enhancement around the orbit	Intravitreal cefazolin and gentamicin. IV ceftriaxone for 4 weeks	No	Recurrent infection despite IV and intravitreal antibiotics	Enucleation (NA)
	2 [*Suwan* et al.^5^]*	58 M	DM	NA	Decreased vision, proptosis, diarrhea and fever.	HM	Significant proptosis (9 mm), eyelid erythema and edema, RAPD, EOM restriction, severe AC inflammation, mature cataract, yellow subretinal mass	Ocular B-scan: dome shaped lesion. CT scan: orbital cellulitis, no abscess, no cavernous sinus abnormalities	Initial IV ceftazidime and cloxacillin switch to IV ceftriaxone once organism identified. No initial Tap/inject. Following PPV, second intravitreal injection vancomycin and ceftazidime	Yes (NA/NA) with intravitreal injection of vancomycin and ceftazidime	–	LP(NA)
	3 [*Davies* et al.^*6*^] *	70 F	Hypertension	Klebsiella pneumoniae liver pyogenic abscess	NA	HM	RAPD, EOM restriction, 1 mm proptosis and 3 mm inferior globe displacement. Eyelid edema, conjunctival chemosis, 1 + AC cells. No fundus view.	MRI: Superior and anterior orbital infiltrate, without involvement of intraconal space	Intravitreal amikacin and cyclosporine. IV ceftriaxone then IV ertapenem and vancomycin. IV methylprednisolone (100 mg daily, total dose 1.4 g). Topical moxifloxacin and cyclopentolate.	No	Dense cataract	LP (1 month)
	4 [*Ghiam* et al.^*11*^]	34 M	Type 1 DM, IVDU	Presumed skin abscesses	Progressive ocular pain	LP	Ptosis, EOM restriction, OHT, eyelid erythema and edema, chemosis, fibrin over the pupil, 0.5 mm hypopyon. No fundus view.	Ocular B-scan: hyperechoic material in the vitreous consistent with vitritis. CT scan: eye proptosis with orbital fat stranding focused primarily around the globe.	Two intravitreal injections (vancomycin/ceftazidime then Only ceftazidime). IV vancomycin and piperacillin/tazobactam then IV cefepime. Topical antihypertensive drops	Yes (NA/ dense vitritis, widespread retinal necrosis, RD)	Refractive high IOP and significant proptosis requiring lateral canthotomy and cantholysis. Despite PPV, NLP and increase ocular pain.	Evisceration (day 9)
**Endophthalmitis with optic neuritis**	5 [*Chiba* et al.^*7*^*]*	79 F	NA	Klebsiella pneumoniae liver pyogenic abscess	Eyelid swelling, severe hyperemia and purulent conjunctival discharge	NA	Hypopyon, cataract	CT scan: nasal scleral rupture and orbital cellulitis. MRI: optic neuritis and ventriculitis	NA	NO	Diseased (day 46)	Enucleation (NA)
**Isolated optic neuritis**	6 [Lee and al.^13^]	56 F	–	Klebsiella pneumoniae liver pyogenic abscess	Decreased vision	LP	RAPD	MRI: enhancement of the left optic nerve	At least 3 weeks of IV ceftriaxone and moxifloxacin. IV dexamethasone (20 mg/day) followed by oral prednisone	No	Small microabscesses in the left frontal and temporal lobes	LP (4 months)

*Although it was not explicitly described, Suwan et al. and Davies et al. described a RAPD which could represent either an extent retinal involvement or concomitant optic neuritis.

## Case report

**Fig. 1:**
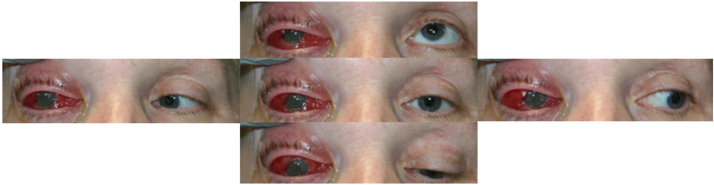
Extraocular movements 48 h following intravitreal antibiotic injection. Right eye showed important eyelid edema and erythema as well as important conjunctival erythema and chemosis. Extraocular movements were limited and painful in all directions*.*

A 59-year-old woman known for a well-controlled type 2 diabetes mellitus with oral medication and prior bariatric surgery presented to a local community hospital with a two-day history of decreased visual acuity, periorbital edema, photophobia, proptosis and pain of the right eye. She was phakic bilaterally, had no prior medial nor surgical ocular history and no previous ocular trauma. Apart from her diabetes and prior bariatric surgery, she had no other risk factors for endogenous endophthalmitis (intravenous drug use, indwelling catheter, immunosuppression). She was seen by the local ophthalmologist who suspected a panuveitis with a hypopyon. The initial empiric treatment decision, awaiting further consultation at our tertiary hospital was oral cotrimoxazole (800 mg/160 mg twice a day) for possible toxoplasmosis, valacyclovir (1 g three times a day) for herpetic acute retinal necrosis and prednisolone acetate (Pred Forte) 1% drops hourly. The acute nature of the presentation with the presence of a hypopyon suggested an endogenous endophthalmitis, but from a yet unknown underlying source. A computerized tomography (CT) scan was performed and no sign of periorbital nor orbital cellulitis was initially seen. She was transferred to our tertiary hospital for further care. At our center, the initial visual acuity was hand motion on the right eye and 20/30 on the left. The presentation with painful ophthalmoplegia (Fig. 1) and a right relative afferent pupillary defect led to suspect concomitant orbital cellulitis (not only preseptal), cavernous sinus involvement or apex syndrome. In diabetics especially, cavernous sinus involvement can occur (other etiologies need to also be considered: ischemic, other infectious agents such as mucormycosis).

**Fig. 2:**
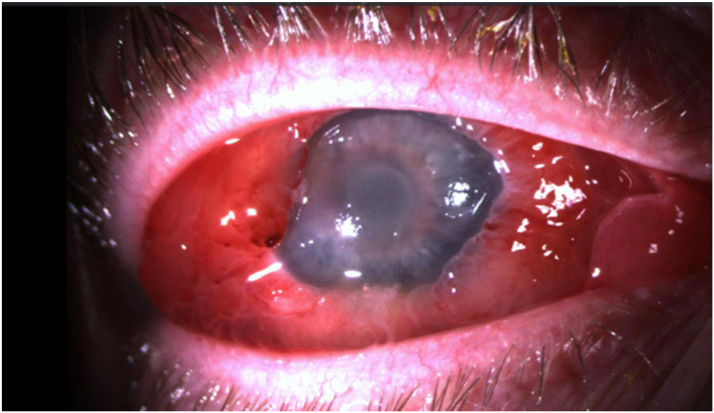
Right eye 48 h following intravitreal antibiotic injection: conjunctival chemosis and erythema, corneal edema, cataract with fibrin on the anterior capsule, 3–4+ cells in the anterior chamber with a 1.5 mm hypopyon*.*

Further, there was erythema, warmth and edema of the right eyelids, and associated ptosis. Slit-lamp exam showed important chemosis and hyperemia of the conjunctiva, corneal edema, 3–4+ cells (according to the Standardization of Uveitis Nomenclature grading) and 2+ flare in the anterior chamber with a 1.5 mm hypopyon. A fundus exam was not feasible due to the anterior chamber findings, cataract and important vitreous haze. A B-scan ultrasonography showed dense heterogeneous intravitreal cellular debris suggesting the presence of significant vitritis. Her blood cultures done the day before had grown *Klebsiella pneumoniae* in line with the diagnostic impression of an endogenous endophthalmitis. A vitreous tap was performed with injection of ceftazidime 2.25 mg/0.1 mL and moxifloxacin 500 mg/0.1 mL. The vitreous culture confirmed *K. pneumoniae* as the causative organism, which was resistant to ampicillin but sensitive to ceftazidime, ceftriaxone, ciprofloxacin, cotrimoxazole and piperacillin/tazobactam. The minimal inhibitory concentrations were ≤0.25 mg/L for ceftriaxone, ≤ 0.06 mg/L for ciprofloxacin and ≤ 2.0 mg/L for amoxicillin-clavulanic acid. The patient was hospitalized, and further questioning revealed a suprapubic discomfort and a fever spike (38.8 ^o^C) one week before hospitalization. On admission, inflammation markers were elevated with a white cell count (WBC) of 9.2 × 109/L and a C-reactive protein (CRP) of 74 mg/L. An abdominal CT scan showed the presence of an acute pyelonephritis secondary to a partially obstructive nephrolithiasis; no liver abscess was seen. The patient was treated empirically with meropenem 2 g IV every eight hours and PF 1% drops were continued hourly (Fig. 2).

Indeed, despite early interventions and subjective improvement of the patient’s symptoms, her visual acuity on the right eye continued to deteriorate to no light perception (LP) within 72 h following her presentation. Repeated imaging of the orbits (CT and MRI) showed signs of right eye panophthalmitis with associated papillitis and orbital cellulitis, but no abscess collection, no sinusitis and no cavernous sinus involvement. Additional daily B-scan ultrasonography evaluations revealed a swollen optic nerve head, a thickened choroid, choroidal detachment, a densely organized vitreous and a possible retinal detachment. Given the clinical deterioration, oral prednisone (30 mg daily) was initiated and a core pars plana vitrectomy (PPV) was performed in order to debride the infectious and inflammatory load, with repeat antibiotic injections (vancomycin 1 mg/0.1 mL and ceftazidime 2.25 mg/0.1 mL) and dexamethasone (400 mg/0.1 mL).

Early postoperative course was favorable, with improved visual acuity to light perception, diminished periorbital edema and pain and decreased signs of cellulitis. According to antibiotic sensitivities, intravenous meropenem was tailored to intravenous ceftriaxone (2 g IV BID) for 3 days and then to oral ciprofloxacin (500 mg twice a day) for a 14 days antibiotic course. The last four days, ciprofloxacin was changed to amoxicillin-clavulanic acid (875 mg PO BID) because of nausea and vomiting. Her oral prednisone (30 mg daily) was tapered. One week following her PPV, proptosis was resolved, extraocular movements were complete and inflammatory markers normalized (WBC: 7.1 ×109/L and CRP: 2.4 mg/L). She was assessed by the urology service and double J-stent placement was planned on an outpatient basis. During the intake of oral prednisone, her glycemia mildly increased which was controlled with increase of her metformin dosage.

Two weeks following her PPV and five days after discontinuing antibiotics, she presented with recurrent periorbital edema and pain with extraocular movement. Visual acuity decreased from LP to no light perception, slit lamp exam showed flare in the anterior chamber with no hypopyon. B-scan ultrasonography showed an organized vitreous with an inferior choroidal detachment. Given the potential diagnosis of either recurrent infection or inflammation, oral ciprofloxacin (500 mg twice per day) was restarted and her oral prednisone (5 mg daily) was increased (10 mg daily). The next day, her clinical presentation worsened with increased erythema, periorbital edema and ocular pain. Her examination showed still trace of cells in the anterior chamber, 2 + of flare, no hypopyon but the new presence of anterior scleritis, and a rise of the CRP (4.4–28.9 mg/L). Ciprofloxacin was changed to intravenous meropenem (2 g IV three times a day), oral prednisone was increased to 40 mg daily and a second PPV was planned. Unfortunately, despite intravenous antibiotics, the patient rapidly developed a globe rupture at the previous vitrectomy trocar incision site with pustular discharge. An emergency evisceration was performed and showed a large inferior subretinal abscess, purulent vitreous and scleral necrosis. The vitreous culture was negative. Meropenem was changed to ceftriaxone (2 g IV daily) five days later, for a total of 12 days following her evisceration, and she remained stable until discharge. The dosage of ceftriaxone was decreased post-evisceration as it was considered as a soft-tissue infection.

## Discussion

Klebsiella endogenous panophthalmitis or endophthalmitis with associated orbital cellulitis are rare and have a dismal visual and anatomic prognosis despite treatment [3], [4], [5].

*K. pneumoniae* virulence is partly explained by its production of a polysaccharide capsule, with some serotype (particularly serotype K1 or K2) offering a higher resistance to phagocytose by neutrophils, especially in poorly controlled diabetics [8]. The hypervirulent *K. pneumoniae* is mostly associated with pyogenic liver abscesses (68%) followed by urinary tract infections (13%) [4], [9]. Metastatic complications, such as endogenous endophthalmitis are highly prevalent compared to systemic infections caused by other organisms (7% versus < 1%) [9], [10]. Although highly prevalent in Eastern Asia and rare in Western countries, some reports have described an increase in the incidence of Klebsiella endogenous endophthalmitis [11].

As illustrated with this case, diabetes is a significant risk factor for developing ocular infection from Klebsiella bacteremia and is also a poor visual prognostic factor. Other published poor prognosis factors include concomitant immunosuppression, a presenting visual acuity of less than counting fingers, delayed diagnosis and treatment, the presence of an hypopyon, rapid onset of symptoms, panophthalmitis or orbital cellulitis, many of which were present in our patient [12].

**Fig. 3:**
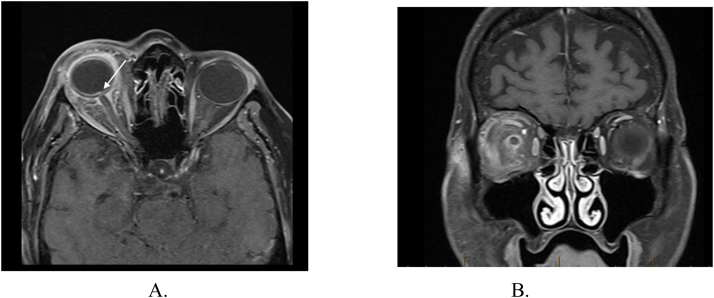
Orbital MRI in the axial (A) and coronal (B) T1- SEFS post-gadolinium administration sequence, showing a right panophthalmitis with associated optic neuritis (papillitis) and orbital cellulitis. Diffuse enhancement of sclera and ciliary body, vitreous heterogeneity with diffuse enhancement pre-septal and post-septal orbital fat was noted, with important conjunctival edema. Enhancement of the optic nerve head (white arrow) of the retrobulbar optic nerve sheath. Retrobulbar optic nerve itself was normal. No signs of an orbital collection, cavernous sinus syndrome or orbital apex syndrome was seen. SEFS: Spin echo fat suppressed.

Our patient presented with classical signs and symptoms of both orbital cellulitis and endophthalmitis which include decreased visual acuity, ocular pain, limited extra-ocular movement, conjunctival chemosis and erythema, diffuse anterior chamber reaction with a hypopyon and diffuse vitritis which is comparable to the other cases described in the literature (Table 1) [3], [4], [5], [7]. Other specific characteristics of Klebsiella endogenous endophthalmitis is a rapid evolution course, production of a subretinal abscess, spontaneous globe rupture and bilateral involvement (13%) [13]. This case shows how virulent this pathogen is. Indeed, no significant pathology of the orbits was visible on the initial CT scan, 24 h before the patient was transferred to our tertiary hospital. Furthermore, early orbital MRI performed 3 days after transfer showed evidence of papillitis with retrobulbar nerve sheath involvement (Fig. 3). There are two reports describing optic neuritis, with or without endophthalmitis nor orbital cellulitis from a Klebsiella pyogenic liver abscess [6], [14]. In the best of our knowledge, this is the second described case of endogenous Klebsiella panophthalmitis with orbital cellulitis and optic neuritis. In the setting of orbital cellulitis, optic nerve involvement can be caused by inflammatory infiltration, mechanical compression by an orbital abscess, ischemia secondary to compression of feeding vessels or dissemination of infection (septic optic neuropathy) [15]. MRI findings include orbital abscess, optic nerve T2-hypersignaling in the setting of optic neuritis, perineuritis can be seen as optic nerve sheath thickening and enhancement in contrast-enhanced fat-suppressed T1-weighted sequence [16]. In B-scan ultrasonography, retrobulbar optic nerve thickening has been reported in both papillitis and retrobulbar optic neuritis whereas optic disc swelling is only seen in papillitis [17]. In retrospect, the finding of an inferior choroidal detachment in the B-scan prior to the evisceration correlates with the sub-retinal abscess found intraoperatively. Therefore, we recommend being alert for choroidal detachment in Klebsiella endogenous endophthalmitis, as it could represent a subretinal abscess.

In this case, orbital MRI showed signs of anterior neuritis with retrobulbar perineuritis (optic sheath enhancement) and no signs of retrobulbar optic nerve involvement or orbital abscess were seen. Those findings could be secondary to either inflammatory changes or infectious dissemination.

Furthermore, bacterial infectious optic neuropathies are not typically associated with common pathogens of endophthalmitis such as Klebsiella, Staphylococci or Streptococci, but are more seen in the setting of tuberculosis, syphilis, rickettsioses and brucellosis [18].

In this particular case, the patient had no other systemic symptoms apart from mild suprapubic discomfort. It reiterates the challenge of suspecting endogenous endophthalmitis without obvious systemic infection, which can lead to delay in treatment [13] In fact, around 25–33% bacterial endogenous endophthalmitis might have a delay in establishing the diagnosis with an estimated average of 3 days delay [19].

Management of all endophthalmitis includes intravitreal antibiotics injection and some patients may require a vitrectomy. Some reports have suggested that early PPV for cases with Klebsiella pneumonia endophthalmitis might improve visual outcomes [13]. Vitrectomy is also associated with a smaller rate of evisceration or enucleation [10]. Multiple intravitreal antibiotic injections may be required as optimal antibiotic intravitreal concentration only lasts 24–48 h following the injection, but repeated injections can be limited by other factors such as retinal toxicity or risk of retinal detachment [20].

**Fig. 4:**
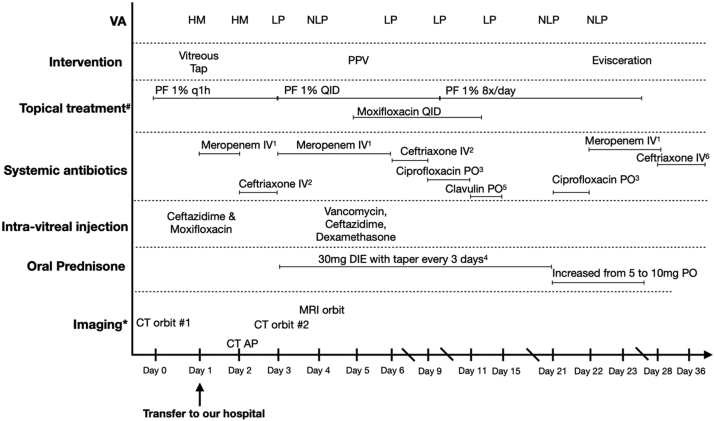
Summary of medical and surgical management of our case ^#^ Topical anti-hypertensive drops were used during all the follow-ups. *A B-Scan ocular ultrasound was performed almost daily. PPV: pars plana vitrectomy. PF: Pred Forte. IV: intravenous. PO: per os. AP: Abdominal-Pelvic. DIE: daily. BID: two times per day. TID: three times per day. QID: four times per day. ^1^ Meropenem 2 g IV TID (on day 2, meropenem was switched for ceftriaxone for less than 24 h and was reintroduced). ^2^Ceftriaxone 2 g IV BID. ^3^Ciprofloxacin 500 mg PO BID. ^4^ Prednisone per OS. ^5^ Amoxicillin-clavulanic acid 875 mg PO BID. ^6^Ceftriaxone 2 g IV DIE. Please refer to the text for information regarding radiological findings*.*

Management of endogenous endophthalmitis also includes systemic antibiotics and the duration depends on the cause of the bacteremia [10]. Management of orbital cellulitis includes systemic antibiotics for at least 2–3 weeks and can be ceased when all clinical signs of orbital cellulitis has been resolved [15] (Fig. 4).

The visual outcome of endophthalmitis depends both on the virulence of the causative organism and on the time of initiation of the therapy. For Klebsiella endophthalmitis, unfortunately, the visual outcome is generally poor. Previous series have shown that 44–69% of eyes with Klebsiella endogenous endophthalmitis have a final VA less than counting fingers and 16–40% of eyes require an evisceration or enucleation [1].

## Conclusion

Klebsiella endogenous endophthalmitis is a devasting ocular disease with poor visual and anatomical outcomes despite early treatment. This case describes a rare presentation of Klebsiella endogenous panophthalmitis with concomitant optic neuritis and orbital cellulitis. We suggest that patients with known Klebsiella infection reporting with any ocular symptoms should be evaluated by an ophthalmologist given the high rate of endophthalmitis and the rapid progression. Multidisciplinary management is essential and early vitrectomy should be performed when possible. Further, in the case of KEE, clinicians should be alert for subretinal abscess when choroidal detachment is observed on B-Scan. In the case of concomitant orbital cellulitis, extended treatment should be considered with an extended course of antibiotics of at least 3 weeks.

## Declaration of funding

None to declare.

## Declaration of conflict of interest

None to declare.

## CRediT authorship contribution statement

**Soumaya Bouhout:** Review of the literature, Retrospective chart review, Writing – original draft. **Magaly Lacrouse:** Review of the literature, Retrospective chart review, Writing – original draft. **Marie-Josée Aubin:** Supervision, Written – review & editing. **Annie-Claude Labbé:** Supervision, Written – review & editing.
